# Patient's families in the ICU: describing their strategies to face the situation

**DOI:** 10.1186/cc9947

**Published:** 2011-03-11

**Authors:** KK Borges, MG Mello, CM David

**Affiliations:** 1 Universidade Federal do Rio de Janeiro, Brazil

## Introduction

The ICU is an environment that generates permanent anguish in family patients due to the possibility of the death of the patient [1]. The stressful situation might induce families to call upon strategies of facing different levels and intensities to keep the harmony of its own emotional structure. The objective is to describe the strategy processes used by families of severely ill patients in the ICU to face the situation.

## Methods

A prospective study covering 14 families. We applied a qualitative method of interviewing and observing participants, complementing the data-gathering by applying the Strategies Inventory of Coping, by Folkman and Lazarus, adapted by Savoia and colleagues [2]. The mixed method used to interpret the results combines the quantitative and qualitative data into only one phase of the study, prioritizing the descriptive-analytical logic. Among the criteria of inclusion are: one member of the patient family in the ICU for more than 1 week, being an adult, must be present in most of the visit periods and receiving physician's information of the patient conditions.

## Results

Families utilize diverse strategies and at different levels, but the most used strategies almost always and most of the time are: escape and avoid (93%), positive re-evaluation and a strategy of problem-solving (79%), social support (43%) and responsibility acceptance (7%). The strategies were considered nonadaptative and the less used were distance, confronting and self-control.

## Conclusions

Escaping and avoidance were the most used due to religious aspects, expressed through perseverance and optimistic attitudes as a way to solve problems, which is directly related to responsibility acceptance and self-control. Positive re-evaluation looks for significance and encouragement to overcome adversity and maintain hope. Knowing such psychological recourses allowed the hospital team to identify the necessity for human assistance to families, making them available in the relationship and prepared in the art of communication.

## References

[B1] Azoulay E Pochard F Chevret S Impact of a family information leaflet on effectiveness of information provided to family members of intensive care unit patients Am J Resp Crit Care Med 2002 165 438 442 11850333 10.1164/ajrccm.165.4.200108-006oc

[B2] Savoia M Santana P Mejias N Adaptação do Inventário de Estratégias de Coping de Folkman e Lazarus para o Português 1996 7 São Paulo: Psicologia USP 183 201

